# Computed tomography radiation doses for common computed tomography examinations: a nationwide dose survey in United Arab Emirates

**DOI:** 10.1186/s13244-020-00891-6

**Published:** 2020-08-03

**Authors:** Mohamed M. Abuzaid, Wiam Elshami, Huseyin Ozan Tekin, Hatem Ghonim, Mona Shawki, Dina H. Salama

**Affiliations:** 1 Department of Medical Diagnostic Imaging, College of Health Sciences, University of Sharjah, P.O.Box 27272, Sharjah, UAE; 2 College of Medicine, University of Sharjah, Sharjah, UAE; 3 Faculty of Medicine, Alexandria University, Alexandria, Egypt; 4 National Center of Radiation Research and Technology, Atomic Energy Authority, Cairo, Egypt

**Keywords:** Computed tomography, Diagnostic reference levels, CT dose indices, CTDI_vol_, DLP

## Abstract

**Objectives:**

Computed tomography (CT) scanning is an essential part of diagnostic and treatment plans, providing swift and accurate diagnostic images. The aim of this study is to develop diagnostic reference levels (DRLs) for the adult common CT examination in the United Arab Emirates (UAE).

**Methods:**

This study presents results of the survey of CT dose indices. The data were collected from 91% of the scanners registered at the Ministry of Health and Prevention (MOHAP) for five common examinations: head, chest, and abdomen-pelvis with and without CM.

**Results:**

CT dose index, dose-length product, and patient weight were analyzed; the reference dose was calculated on the 75th percentile, and an achievable dose was proposed from the median value. The results were compared with the UAE initial National Dose Report as well as the international reports.

The proposed dose for CTDI_vol_ (mGy) and DLP (mGy cm) is as follows: head without CM 40 and 695, head with CM 48 and 820, chest 10 and 275, abdomen-pelvis without CM 14 and 810, and abdomen-pelvis with CM 20 and 1025.

**Conclusions:**

The results show low dose variations between the MOHAP scanners. The data also revealed CTDI_vol_ and DLP values comparable to those in the initial NDRL report and international standards. The establishment of diagnostic reference levels will require a continuous dose monitoring system.

## Key points

- DRLs for adult CT examinations have been investigated in the UAE.
- Data included age, sex, weight, dose indices to include CTDI_vol_, and DLP.
- The DRL for CTDI_vol_ is comparable to other countries.
- The results indicated that there is potential for dose optimization.

## Introduction

In 1996, the International Commission of Radiation Protection (ICRP) first introduced diagnostic reference levels (DRLs) [1]. The aim of this measure was to manage the appropriate patient dose for the imaging procedure’s clinical purpose. DRLs are a tool used to identify situations in which radiation dosage is unusually elevated [2]. The establishment of DRLs is considered the initial step in the radiation dose monitoring process [3].

Optimization of CT examination through the comparison of the patient doses with DRLs is recommended by the International Authority (IAEA), IRCP, and European Commission (EC) [2]. These organizations encourage individual countries to establish their local DRLs and to conduct continuous monitoring for the DRL values. ICRP recommended that each country surveys the medical imaging practice to determine national DRLs to be used as indicators, provide guidance for dose optimization, and ensure justification of appropriate dose for a given clinical indication [4, 5].

The UAE’s Federal Authority for Nuclear Regulation (FANR), in Regulation 24 (version 1), defines the DRL as “a level used in medical imaging to indicate whether, in routine conditions, the dose to the patient or the quantity of radioactive material administered in a specified radiological procedure is unusually high or low for that procedure” [6]. In 2014/2015, FANR, in collaboration with IAEA, initiated a project aimed at establishing NDRLs. The hospital and health organizations that participated were Dubai Health Authority and Abu Dhabi Health Service hospitals. DRL data for dental radiography, mammography, CT, and nuclear medicine were obtained [7].

In 2017, initial NDRLs were endorsed by the FANR Radiation Protection Committee for use and implemented in the UAE healthcare facilities. To establish NDRLs, initial DRLs are set to be the baseline for healthcare providers (both private and governmental) to develop their facility DRLs. The initial DRLs covered four CT scan examinations with the brain (871 mGy cm), brain with contrast (1071 mGy cm), chest (443 mGy cm), and abdomen/pelvis (671 mGy cm) [7]. As this is an initial report, it covered only the DLP data, requesting all healthcare organization to establish their DRLs, which will be analyzed to establish NDRLs.

We surveyed the radiation doses administered to patients in terms of the CT dose index (CTDI_vol_) and dose-length product (DLP). The study’s primary goal was to collect CT dose index data from MOHAP CT scanner facilities to contribute to the establishment of national DRLs for CT examinations commonly performed on adult patients. Furthermore, the survey will provide a current snapshot of CT equipment technology and CT imaging practices in the UAE.

## Materials and methods

### Demographics and facility selection

Eleven CT scanners are currently available in MOHAP hospitals. The inclusion criteria were availability to participate in the survey, registration with the regulatory authority, and a workload of more than 100 CT procedures per month. An invitation was sent to each facility, and ten CT scanners out of the total eleven invited fulfilled the requirements for enrolment in the study. Only one scanner was excluded from the study as it was in service during the data collection period.

### Survey design and data collection

The survey used previously (2017) in a CT dose survey performed in Egypt in collaboration with IAEA, which was kindly shared by the author. The data collected comprise scanner information, routine CT protocols, patient weight, examination data, and dose indices (CTDI and DLP) [3].

Data were collected from each scanner by selected individuals from the same facility. They all worked in the same place and had relevant experience; they had also attended a brief education session to pinpoint the importance of collecting high-quality data and how best to achieve this. The survey distributed between the CT scanners that took part in the study, and the results obtained were extracted using MS Excel templates. Each hospital was given 16 weeks to complete the survey. The number of cases for each procedure in previous studies and recommendation varied from 10 to 20 patients [8, 9]. In this study and for better typical practice, 20 patients from each procedure were selected. The hospital statistics identify five common examinations, and the highest frequency CT examinations performed as follows: CT head with and without contrast medium (CM), chest without CM, and abdomen-pelvis with and without CM. In this study, the obtained data used the displayed DLP and CTDI_vol_. The final data was checked for errors prior to analysis.

### Statistics analysis

All data were organized using Microsoft Excel with each body procedure dealt with in a separate worksheet. Data for CTDI_vol_, DLP, and patient weight were analyzed for each exam with respect to minimum, maximum, and standard deviation. Additionally, the first quartile (25th percentile), second quartile (median, 50th percentile), and third quartile (75th percentile) values were calculated. The mode was calculated as a typical value (i.e., the most repeated in the data set). The achievable dose was proposed based on the median value of CTDI_vol_ per sequence and DLP per examination. The data used to establish DRL at the hospitals were based on the rounded third quartile and compared with the initial NDRL report as well as the available international reports [3, 10, 11].

### Ethical approval

The MOHAP research ethics committee approved the study (reference number MOHAP/REC-18/2018). All methods and study protocols implemented were in accordance with the relevant guidelines and regulations. Patient consent requirements are waived for retrospective data collection.

## Results

### CT scanners and survey sample

The authors worked closely with the radiology department to promote and complete the data collection and ensure that a representative sample was polled. Ten out of the eleven machines (91%) registered at MOHAP hospitals were included in the survey. One scanner was excluded from the study as it was out of service during the data collection period.

All the scanners installed in the participating hospitals were manufactured by General Electric (GE). Table 1 presents information on the scanner models, number of detectors, kVp, mAs, rotation time, and dose modulation. All machines were manufactured between 2008 and 2014 and installed between 2014 and 2017. All CT scanners were calibrated prior to data collection to ensure the displayed values such as CTDI_vol_, mAs, DLP, kVp, slice thickness, and CTDI_w_ were calibrated correctly.

**Table 1:** Scanner information including scanner model, number of the detector, kVp, mAs, dose modulation, and rotation time

Model	No. of scanners	No. of detectors	kVp (min–max)	mAs (min–max)	Dose modulation, yes/no, type	Rotation time
Revolution Evo	9	128	80–140	80–500	Yes, ASIR	0.35–1.0
Discovery CT750	1	64 dual	80–140	80–800	Yes, ASIR	0.35–2.0

### Patients’ characteristics

Data were collected for a total of 940 patient procedures. Two hundred patients were represented by CT of the head with CM, head without CM, chest, and abdomen-pelvis without CM from 10 scanners, and 140 patients were represented by CT of the abdomen-pelvis without CM from seven scanners.

The weight of the patients included in the study ranged from 40.0 to 123.0 kg, with medians for different examinations between 76.0 and 80.0 kg. The median distribution of weight in different scanners ranged from 75.0 to 80.8 kg, and they ranged between 56.0 and 97.0 kg. The patient weight included in this study as a parameter may affect the patient dose; therefore, instead of assuming that all the adult patients have an average size, patients’ average size considered was based on the median of individuals in different procedures (76–80 kg) with a range between 39 and 123 kg. In African and Asian countries, individuals’ weight of 60 kg is considered average and 70 kg in developed countries. Although the UAE is a part of the Asian countries, it is ranked fifth in global obesity. This fact in addition to the used protocol can explain the higher CTDI value, and in the future study and with the protocol optimization and clinical indication DRL establishment, patient weight will be better controlled [7, 8].

### CT parameters

Table 2 shows the examination protocols in the surveyed hospitals. The use of scanners from a single manufacturer (GE Company) ensures the similarity of the protocols used. The protocols used throughout scanner were optimized after the scanner installation (2014–2017). Protocol optimization was conducted by senior radiologists, CT technologists, and the clinical specialists to consider adult and pediatric protocols. Clinical indication-based and patient size protocols were not included in this stage of study.

**Table 2:** Exposure parameters (number of series, tube voltage, tube current to time product, rotation time, and pitch) per examination type

CT protocol	kVp, min–max (mode)	mAs, min–max (mode)	Rotation times, min–max (mode)	Pitch, min–max (mode)	Number of Series
Brain without CM	100–135 (120)	100–430 (100)	0.5–1.0 (0.5)	0.765–1.5 (1.0)	1
Brain with CM	100–135 (120)	100–430 (100)	0.28–1.0 (0.5)	0.765–1.5 (1.0)	2
Chest	100–135 (120)	100–420 (100)	0.5–1.0 (0.5)	0.765–1.5 (1.0)	1
Abdomen-pelvis without CM	100–135 (120)	100–430 (100)	0.28–1.0 (1)	0.2–1.5 (1.0)	1
Abdomen-pelvis with CM	100–135 (120)	100–430 (400)	0.5–1.0 (0.5)	0.765–1.5 (1.0)	2

Description of the exposure parameters’ minimum, maximum, and mode was used for different examinations: tube voltage (kV), milliampere-seconds (mAs), rotation time(s), and pitch. The data were collected from the helical scans only, which are used as the routine protocol for all procedures.

Head scan data were performed with a single-phase when no indication for contrast media injection is present and when contrast media are used. Data were collected from one sequence for chest and abdomen without contrast and two sequences for abdomen-pelvis with contrast.

GE scanner was equipped with ASIR image reconstruction algorithm which leads to radiation dose reduction of up to 27% without degrading the image quality [9].

### Patient dose indices

Table 3 shows a statistical analysis of the radiation dose index in terms of CTDI_vol_ per sequence and DLP per examination. The examination CTDI_vol_ and DLP median values were used as representative of typical practice. The table also includes the CTDI_vol_ and DLP minimum–maximum range, ratio, mean, median, and first and third quartile values of CTDI_vol_ and DLP.

**Table 3:** Analysis by examination type of average CTDI_vol_ per sequence and total DLP per examination, for distribution of median values per CT scanner

CT protocol	Median values of CTDI_vol_ per sequence					Median values of DLP per examination				
	Range, min–max (max/min ratio)	1st quartile (25%)	Median (50%)	Mean	3rd quartile (75%)	Range, min–max (max/min ratio)	1st quartile (25%)	Median (50%)	Mean	3rd quartile (75%)
Head without CM	6.4–54.7 (8.6)	27.2	29.9	32.1	39.6	384.8–743.0 (1.9)	491.4	639.3	592.7	693.1
Head with CM	25.0–54.7 (2.2)	34.6	41.4	41.1	48.0	430.0–1168.0 (2.7)	676.4	772.2	780.1	818.7
Abdomen-pelvis without CM	4.4–15.7 (3.6)	9.1	11.0	10.9	13.5	330.0–1186.8 (3.6)	551.1	656.2	683.2	811.8
Abdomen-pelvis with CM	11.3–25.0 (2.2)	13.3	16.9	17.2	20.4	363.0–1698.0 (4.7)	533.6	606.5	825.9	1023.1
Chest	4.7–12.3 (2.6)	5.6	7.1	7.8	10.2	137.5–360.8 (2.6)	227.2	251.7	255.0	276.2

### CT dose index and DRL calculation

The initial proposed DRLs and achievable doses for the CT examinations are listed in Table 4. DRL median CTDI_vol_ and DLP were used to estimate the typical dose in each scanner. The proposed DRLs for MOHAP scanners were set based on the 75th percentile values of medians for the median CTDI_vol_ and DLP from each scanner. Achievable dose was proposed from the medians (50th percentile) of CTDI_vol_ and DLP value per examination [10]. Comparison between the recently published DRLs in the UAE and those of other countries is presented in Table 5.

**Table 4:** Initial DRLs and achievable doses for CT examinations in MOHAP hospitals

CT protocol	MOHAP DRL		Achievable dose	
	CTDI_vol_/sequence, mGy	DLP/examination, mGy cm	CTDI_vol_/sequence, mGy	DLP/examination, mGy cm
Head without CM	40	695	30	640
Head with CM	48	820	41	770
Abdomen-pelvis with CM	14	810	11	655
Abdomen-pelvis without CM	20	1025	17	605
Chest	10	275	7	250

**Table 5:**
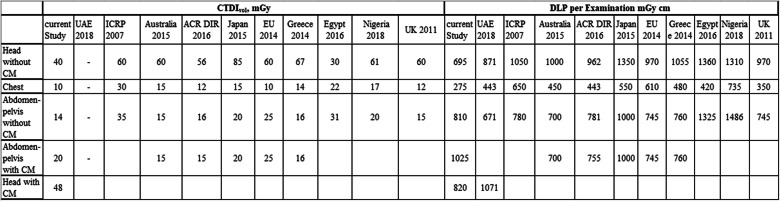
Comparison between THE current study, FANR, and international studies

## Discussion

This is a large-scale study that will contribute to the establishment of national DRLs in the UAE. It includes a description of the methodology, approaches used, and instruments employed, which covers a wide geographical area, routine CT examinations, patient characteristics, dose indices, and comparison between the initial NDRLs and those of other countries.

This study describes a preliminary dose index for CTDI_vol_ and DLP from the scanners belonging to MOHAP hospitals in the UAE. The dose index will be used to establish the local and national DRLs and as a tool for radiation dose monitoring and minimization whenever high dose levels are identified.

A limited number of studies have been published on dose indices from the UAE. The initial NDRL report published by FANR in 2018 includes four procedures: head with and without contrast, chest, and abdomen without contrast with the DLP dose only. The report covers two of the seven emirates that make up the UAE. Comparison of this study’s results with the FANR report revealed that the DLP values in this study were lower in three procedures—head without CM, head with CM, and chest—and higher in the abdomen without CM.

CT dose indices in the region were available from Egypt [12], Sudan [13], Saudi Arabia [14], and Bahrain [15], with limited information from other neighboring countries. The Sudan study included pediatric patients with DLP dose only. The study from Bahrain was limited to one hospital. A study from the western region of Saudi Arabia concentrated on the establishment of DRLs for CT trunk imaging with a minimum of 10 patients from each site. A survey of CT dosage in Syria was conducted in 2009 and thus is relatively old with no updates available.

Table 5 shows a comparison of the CTDI_vol_ and DLP results with those of Australia (2015), ACR DIR (2016), Japan (2015), the EU (2014), Greece (2014), Egypt (2017), and ICRP (2007) [3, 16–18]. The values in this study were approximately lower than and comparable with those reported in other studies.

This study indicated that the main reason that the UAE’s dose index is relatively low or lower than those reported in other studies is the use of standardized protocols. Furthermore, the relatively new scanners were manufactured by a single company (GE), which may lead to substantial homogeneity in the radiation outputs owing to the similar technology and protocol used. Further investigation is required to investigate the acceptability of image quality. The results demonstrate a variation in the CTDI_vol_ dose quantities for the head without CM up to 9-fold in ranged between 6.4 and 54.7 mGy, and for the abdomen without CM up to threefold ranged between 4.4 and 15.7 mGy. Generally, variation in the protocol can affect the radiation dose; therefore, the same scanner might result in doses higher or lower than the DRLs. Similar or higher variation was reported: about 12-fold in the UK and more than 20-fold in the USA, and 2.9- to 10.4-fold variations in the head and abdomen-pelvis examinations [17]. Foley et al. reported variations of up to 89% dose variation using identical scanners in different sites [18]. In this study, the variation did not exceed the NDRLs, which requires further investigation to determine the variability in scanning protocols, the protocol optimization, and the potential effect of dose saving applications such as ASIR.

It has been observed from another study that CT technologists’ practices apply standard protocol for all patients and procedures [19]. The radiation dose received by the patients may be lowered by changing the protocols and exposure factors according to patient size and weight. Training and continuous education programs may address such opportunities and improve technologists’ confidence.

This study is the largest CT dose surveys conducted to date in the UAE, and its results will contribute to the FANR’s efforts to establish the NDRL. The study has established a starting point for dose monitoring efforts in MOHAP CT scanners. High doses may be monitored based on comparison to the study baseline for current procedures. Through continuous engagement with the manufacturers, clinical specialists can improve the protocol. Re-assessment of practice and dose monitoring will improve dose reduction over time. The study results are shared with the involvement of the imaging department at MOHAP for use in the establishment of DRLs among the hospitals’ network.

### Limitation

The accuracy of CTDI and DLP values was not done considering that the values read from the machine are not too different from actual values [11]. Estimation of DRLs based on clinical indication is identified as the future improvement of this study as well as further protocol optimization.

## Abbreviations

CTDI: Computed tomography dose index
DLP: Dose-length product
DRL: Diagnostic reference level
FANR: Federal Authority of Nuclear Regulation
IAEA: International Atomic Energy Agency
ICRP: International Commission on Radiological Protection
IRC: International Radiology Center
kVp: Tube voltage
mAs: Tube current time
NDRL: National diagnostic reference levels
UAE: United Arab Emirates
UNSCEAR: United Nations Scientific Committee on the Effects of Atomic Radiation
WHO: World Health Organization
